# Guillain-Barré syndrome AMSAN variant in a 90-year-old woman after COVID-19: a case report

**DOI:** 10.1186/s12877-023-03833-1

**Published:** 2023-03-01

**Authors:** Chiara Sidoli, Adriana Antonella Bruni, Simone Beretta, Paolo Mazzola, Giuseppe Bellelli

**Affiliations:** 1 School of Medicine and Surgery, University of Milano-Bicocca, U8 Building, Floor 4, Lab 4045, Via Cadore, 48, 20900 Monza, MB Italy; 2 Acute Geriatrics Unit, San Gerardo hospital ASST Monza, Monza, MB Italy; 3 Neurology Unit, San Gerardo hospital ASST Monza, Monza, MB Italy; 4 NeuroMI – Milan Center for Neuroscience, Clinical Neurosciences Research Area, Milan, MI Italy

**Keywords:** Covid-19, Guillain-Barré syndrome, Motor and sensitive polyneuropathy, Geriatric syndrome

## Abstract

**Background:**

Guillain-Barré syndrome (GBS) is an inflammatory disease of the peripheral nervous system characterized by rapidly evolving polyneuropathy caused by autoimmune demyelination and/or axonal degeneration. Since SARS-CoV-2 outbreak, several GBS cases following exposure to coronavirus disease-2019 (COVID-19) have been reported in literature, raising the concern of the latter being a potential trigger event for GBS.

**Case presentation:**

We report the case of a 90-year-old Caucasian woman who was admitted to our hospital because of fatigue, worsening gait and leg strength, dysphonia, dysarthria and dysphagia, started 3 weeks after being exposed to COVID-19. Based on clinical presentation GBS was suspected, so she performed a lumbar puncture and electromyography, which confirmed the diagnosis of acute motor and sensory axonal neuropathy (AMSAN) variant. We administered high dose of intravenous immunoglobulin with slight neurological improvement. However, after 2 weeks of hospitalization with maximization of care, her physical condition worsen, manifesting severe frailty. The patient was discharged with home support services for managing parenteral nutrition and intense scheduled physiotherapy. A few days later, the patient experienced a further decline in her clinical condition and died at home.

**Conclusions:**

To the best of our knowledge, we report the oldest woman with GBS AMSAN variant after COVID-19 described in the existing literature. Our case supports further research aimed at improving recognition, characterization and prompt management of neurological diseases related to COVID-19 in older patients.

**Supplementary Information:**

The online version contains supplementary material available at 10.1186/s12877-023-03833-1.

## Background

GBS is a rare neurological disease in which an autoimmune process leads to myelin disruption and/or axonal degeneration of nerves and roots, causing rapidly progressive weakness in limbs and/or facial muscles [1, 2]. Infectious diseases are often recognized as one of the main trigger cause, and neurological symptoms occur a few days to 5 weeks after the trigger event [3, 4]. A recent meta-analysis shows that the incidence of GBS ranges from 0.62 to 2.66 per 100,000 person every year, with higher risk of developing it in males than in females [3].

The main treatment for GBS consists of immunotherapy with intravenous immunoglobulin (IVIG) or plasma exchange [1]. Most patients have favorable prognosis with resolution of symptoms within weeks or months [5]. Potential residual disability depends on the patient’s pre-existing functional status, the type of GBS, the patient’s response to immunotherapy and intercurrent complications.

Since fall 2019 to date, the world’s health systems have had to deal with respiratory diseases related to SARS-CoV-2 outbreak. However, association with acute neurological illnesses, including GBS, have also been detected [6]. Indeed, since infectious disorders and GBS are intertwined, there is growing concern in the literature that COVID-19 is a potential triggering event for GBS.

This paper contributes to this field of research by reporting a case of the oldest woman with GBS AMSAN-type who was admitted to our hospital after being exposed to COVID-19, a situation previously described in only a few cases, of which a comparative analysis is drawn.

A timeline indicating the clinical and diagnostic workup is available in the supplementary material section (Appendix 1).

## Case presentation

A 90-year-old Caucasian woman was admitted to the Emergency Department (ED) of our hospital on January 12, 2021 due to fatigue, worsening of gait and leg strength, dysphonia, dysarthria and severe difficulty in swallowing, impairing oral intake. All reported symptoms originated in the previous 2 weeks. Due to the initial loss of appetite, the patient previously went to the ED on December 26, 2020 for a check-up and was subsequently discharged after intravenous hydration.

Her medical history consisted of long-term hypertension controlled with ramipril and bisoprolol prescribed by the general pratictioner, who provided her with regular follow-up, and knee bursitis with favorable resolution. Her constitutional status was normal (Body Mass Index 23.4 Kg/m^2^) and she did not suffer from diabetes nor dyslipidemia. Moreover, she suffered from COVID-19 infection in the first part of December, 2020; no oxygen supplementation nor hospitalization was necessary, only paracetamol was administered to treat fever. On December 26, 2020, she performed a SARS-CoV-2 molecular swab test, which was positive.

Before COVID-19 infection, the patient was reported to be autonomous in basic (Katz’s ADL 6/6) and partially dependent in instrumental (Lawton and Brody’s IADL 5/8) activities of daily living. Furthermore, she was able to walk without assistance and had no history of dysphagia before COVID-19 infection. She had no pre-existing cognitive decline nor were history of substances abuse; steroid and opioid intolerance reported without other information. The Clinical Frailty Scale score was 4 (“very mild frailty”).

Upon admission to the ED, her blood pressure was 130/80 mmHg, heart rate 80/min rhythmic, respiratory rate 14 breaths/min and peripheral oxygen saturation was normal. Cardiovascular, thoracic and abdominal examination were unremarkable. Neurologic examination showed normal consciousness, bulbar signs with dysarthria, dysphonia, dysphagia, no facial nerve palsy, mild symmetric weakness in the upper limbs (Medical Research Council [MRC] muscle power score 3/5 in both arms), asymmetric weakness in the lower limbs (MRC score 3/5 left leg, 2/5 right leg), asymmetric sensory signs in the lower limbs (severe hypoesthesia in the left leg, hyperesthesia in the right leg), absent deep tendon reflexes in the lower limbs, normal plantar response.

The patient remained in the ED for 1 day, and she underwent laboratory and radiological investigations, with subsequent specialist medical evaluation. Specifically, brain CT scan showed chronic ischemic changes of the white matter and diffuse cortical atrophy, and a small meningioma in the greater wing of right sphenoid bone without clinical relevance. Laboratory tests revealed elevation of leucocytes (10.540/mmc with 85% of neutrophils), C-reactive protein (2.3 mg/dl) and D-dimer (4976 ng/ml). SARS-CoV-2 molecular swab test at admission and after 5 days of hospitalization were both negative.

Afterward, she was evaluated by both the neurologist and infectious disease specialists, who suspected GBS after COVID-19 infection versus viral encephalomyelitis. Consequently, a lumbar puncture was performed and empiric antimicrobial therapy, consisting of intravenous acyclovir and ampicillin, was initially administered to cover a possible viral and/or bacterial infection.

Later, on January 13, the patient was transferred to our Acute Geriatrics Unit (AGU) in stable conditions. We revealed a 1 cm wide sacral excoriation, which was treated with advanced medical dressing.

In the AGU she continued antimicrobial therapy and started intravenous fluids for dehydration. Cerebrospinal fluid (CSF) analysis showed a mildly raised protein content (57 mg/dl) and cell count 21/microL (morphologically defined as matured lymphocytes). CSF culture was negative. Furthermore, during the second day of hospitalization we performed an electromyography, which evidenced axonal motor-sensitive polyneuropathy (Tables 1 and 2).

**Table 1:** Sensory nerve conduction study

Nerve- position	Velocity (ms)	SAP amplitude (μV)
**L Ulnar** - V **finger**	n.e.	n.e
L **Sural** – **Lateral Malleolus**	n.e.	n.e

**Table 2:** Motor nerve conduction study

Nerve- position	Distal latency (ms)	CMAP distal amplitude (mV)	CMAP proximal amplitude (mV)	Velocity (m/s)
**L Ulnar**				
wrist	2.97	5.3	4.3	
forearm	7.08	5.4	4.3	51
**R Peroneal**				
ankle	3.02	0.1	0.2	
leg	9.43	0.2	0.1	46.8
**L Tibial**				
ankle	3.23	6.9	4.8	
knee	11.56	4.3	2.9	42

*Abbreviations*: *L* left, *R* right, *SAP* sensory action potential, *CMAP* compound motor action potential, *n.e* not elicitable

Based on clinical presentation, electro-neurophysiological and CSF findings, she was assessed by a neurologist, who confirmed the diagnostic suspicion of GBS AMSAN type. Following this, we promptly administered her high dose of IVIG (0.4 mg/Kg/die) for 5 days. No side effects were reported during and after IVIG infusion.

In order to limit malnutrition (serum albumin level: 3.3 g/dl; serum B12 vitamin: 450 pg/mL; serum folate: 1.7 ng/mL; serum iron: 28 mcg/dL; serum ferritin: 39 ng/mL; serum transferrin: 270 mg/dL; transferrin saturation: 5%; serum total cholesterol: 186 mg/dL; serum triglycerides: 120 mg/dl; fasting plasma glucose: 90 mg/dl), she started parenteral nutrition (1200 mosm/L) under dietitian’s prescription, using central venous catheter (CVC). The reason for choosing parenteral instead of enteral nutrition was the patient’s refusal and intolerance to nasogastric tube.

Further radiological study with MRI was not feasible for this patient due to lack of availability of the procedure for service overload.

After 5 days of IVIG therapy, she was evaluated by the neurologist who noted a slight neurological improvement: she was able to speak louder with less dysarthria and could barely move her arms. However, the motor deficit in the lower limbs persisted, particularly in the right leg, impairing the ability to walk. Dysphagia (from both liquid and solid) was unchanged, impairing oral intake.

Autonomic disturbances also occurred. From the second day of admission in AGU, she suffered from uncontrolled hypertension and acute urine retention. Therefore, we optimized the antihypertensive therapy with amlodipine and transdermal clonidine and placed a urinary bladder catheter. Microbial urine sample was negative. Moreover, she developed hypoxemic respiratory failure on the third day of hospitalization. Given the strong suspicion of aspiration pneumonia, laboratory test showed elevated serum C-reactive protein and neutrophils, and chest X-ray confirmed pneumonia in the lower right side. Antimicrobial therapy with metronidazole was performed, resulting in respiratory recovery in the following days.

Despite the maximization of care and the initial neurological improvement, her general conditions progressively worsened. Indeed, blood tests showed a worsening of the nutritional status (serum albumin level: 2.3 g/dl) with a fluctuating vigilance state tending to drowsiness (CT scans was negative for ischemic or hemorrhagic lesions). She also refused passive and active mobilization, compromising potential recovery. Our comprehensive geriatric assessment showed a state of severe frailty related to severe malnutrition, functional and physical disability, urinary incontinence and pressure ulcers, giving her higher risk of adverse events and a poor prognosis.

As the patient wittingly refused to be transferred to a Rehabilitation Unit, she was discharged to her son’s home after 15 days of hospitalization, in a relatively stable clinical conditions but with poor chances of recovery, with home support services for parenteral nutrition management and intensive physiotherapy scheduled. Telemedicine follow-up after 2 months revealed a rapid further decline in her clinical condition, and she died at home a few days after hospital discharge.

## Discussion and conclusions

In this article we provide a detailed report of a geriatric case of GBS AMSAN-type after COVID-19 infection.

According to the Brighton diagnostic protocol, following clinical, neurophysiological and laboratory criteria, the patient fulfilled the Level-1 criteria for GBS diagnosis. In detail, they encompass: patient’s history of infectious disease; weakness of the limbs and cranial nerves that worsens in about 4 weeks reaching a plateau; severe axonal motor-sensitive polyneuropathy confirmed by electromyography; elevate protein count with < 50 cells in the CSF [1, 7]. Furthermore, her neurological signs and symptoms tended to improve after IVIG infusion, despite AMSAN variant being described in literature as one of the most severe GBS forms with a poor response to immunotherapy and an even worse prognosis [4].

The use of IVIG is also widespread in the elderly population, as the risk-benefit ratio in favor of the treatment has been demonstrated, summarized in a systematic review [5]. However, caution should be adopted in patients with renal insufficiency and pro-thrombotic risk factors, since IVIGs increased blood viscosity. This confer a greater risk of developing thromboembolic events, kidney and heart failure [8, 9]. In our patient hypertension was initially poorly controlled by antihypertensive treatments, and we found a possible twofold justification for this phenomenon. The first and most probable cause, is autonomic involvement during the demyelination process. This hypothesis is supported by studies that evidenced axonal demyelination of baroreceptors [10]. Another hypothesis stems from a possible side effect of IVIGs, since hypertension has been reported as an adverse reaction [8, 9]. We therefore assumed that the main cause was autonomic dysfunction due to GBS, probably worsened by IVIG therapy. The authors recommend close monitoring of blood pressure and the search for possible arrhythmias, which are a potentially life-threatening autonomic complication of GBS.

A key aspect of managing the elderly patient with GBS is also the supportive care. This stand on the fact that regardless of specific IVIG therapy, older age is an independent unfavorable prognostic factor, and the presence of additional geriatric syndromes predisposes the patient to a higher likelihood of adverse events with a poor prognosis [11]. Thus, the goal of comprehensive geriatric assessment is to identify potential disabling conditions and implement treatments aimed at improving quality of life [12]. In our case, the patient showed a state of malnutrition, dysphagia and physical disability due to GBS, and a pressure sore due to immobilization. Therefore, we set up supportive therapy with parenteral nutrition, encouraged early mobilization, and medicated the pressure sore with advanced dressings. Unfortunately, she died despite optimized medical therapy, probably due to GBS complication, although no autopsy findings were made. At discharge, the patient had a very poor prognosis for multiple reasons. First, it is due to GBS AMSAN-type, which is one of the most severe forms of GBS [1]. Second, the development of refractory hypertension and the onset of a pulmonary infectious event that debilitated her. Finally, the state of severe frailty due to malnutrition and bed restraint, as well as the presence of the sacral decubitus ulcer and bladder catheter that exposed her to a high risk of infectious events. Despite the patient’s poor outcome, the authors strongly suggest a geriatric multidimensional evaluation to treat geriatric syndromes and ensure a better prognosis for the elderly patient.

Nowadays it remains unresolved how COVID-19 acts as trigger for immune dysregulation. Emerging studies evidenced the neurotrophic properties of Coronaviruses, and in particular of SARS-CoV-2 [13, 14]. In this context, some cases of GBS and its variant after COVID-19 infection have been described (including this one). Furthermore, the severity of SARS-CoV-2 pneumonia and neurological complication has been associated with high serum aldosterone levels, which stimulates IL-6 and cytokine storm production, as described by Campana et al. [15].

According to a recent systematic review, the prevalence of GBS after COVID-19 infection is 0.42% [16], with the AMSAN variant reported in only about 7 of 73 total cases [17]. A detailed comparative analysis is reported in Table 3 [18–23].

**Table 3:** Summary of all GBS AMSAN variant features after COVID-19 exposure

Patient	Age	Sex	Comorbidity	GBS diagnosis	Additional Instrumental Diagnosis	Intercourse between COVID-19 infection and GBS symptoms (days)	IVIG administration	Outcome	
G.A.C.	90	F	Hypertension	Clinical, CSF, EMG	none	30	Yes	Death	
Assini et al. [18]	60	M	None	Clinical, CSF, EMG	Antibody antiganglioside; SARS-CoV-2 in CSF	20	Yes	Persistent hyporeflexia; slight improvement in right foot drop	
El Otmani [19]	70	F	Rheumatoid Arthritis	Clinical, CSF, EMG	SARS-CoV-2 in CSF	3	Yes	No neurological improvement	
Mozhdehipanah [20]	55	F	COPD	Clinical, CSF, EMG	None	26	Yes	Death for ARDS after third day of IVG	
Padroni et al. [21]	70	F	None	Clinical, CSF, EMG	None	23	Yes	Unknown	
Sedaghat et al. [22]	65	M	Diabetes mellitus	Clinical, CSF, EMG	Cerebral and spinal MRI	14	Yes	Unknown	
Toscano et al. [23]	77	F	hypertension, stroke, atrial fibrillation	Clinical, CSF, EMG	Antibody antiganglioside; SARS-CoV-2 in CSF; Cerebral and spinal MRI	7	Yes	No neurological improvement	
Toscano et al. [23]	23	M	None	Clinical, CSF, EMG	Antibody antiganglioside; SARS-CoV-2 in CSF; Cerebral and spinal MRI	10	Yes	Neurological improvement of ataxia and facial weakness	

*Abbreviations*: *CSF* cerebrospinal fluid, *EMG* electromyography, *MRI* magnetic resonance imaging, *CODP* chronic obstructive pulmonary disease

In particular, the similarities are the univocal GBS diagnosis (complete with clinical examination, electrophysiological and CSF test) and the similar treatment with IVIG protocol. The differences range from the patients’ age to the timing between COVID-19 infection and the onset of GBS. To the best of our knowledge, our patient was the oldest among the seven other cases of AMSAN-type, and the second oldest among all 73 GBS cases reported. Secondly, in four patients GBS occurred within 15 days of COVID-19 infection, while in the remaining three patients the onset occurred at least 3 weeks after COVID-19 exposure. In our patient, the period between COVID-19 infection and the onset of GBS was approximately 1 month.

Regarding the GBS diagnosis, although all researchers used the same diagnostic algorithm, a few authors strengthen it with MRI spine, antiganglioside antibodies serum test or SARS-CoV-2 CFS test; the type of supportive test performed may be related to hospital availability. The study of cranial nerves for the early diagnosis of GBS appears to be an interesting novelty. Manganotti et al. highlight deficits in the facial nerve or other cranial nerves during COVID-19 infection, which could anticipate the systemic manifestation of GBS [24]. The authors suggest performing a neurological examination and possibly a diagnostic investigation in patients with ageusia or other cranial nerve-related disorders, in order to promptly diagnose GBS, then administer IVIG earlier and improve chance of recovery.

In another review by Finsterer et al., 220 cases of patients with GBS were collected, of which 11 were affected by AMSAN variant [25]. Similarly, the review highlights the manifestation of post-infectious neurological disease resulting from COVID-19, with the possibility of GBS manifestation also in elders. Again, our case report confirms the exclusivity of the description of the oldest patient with GBS AMSAN variant after SARS-CoV-2 infection.

This is the oldest female patient with COVID-related AMSAN variant of GBS, reported in the current literature. Our case report highlights the importance of early recognition of GBS symptoms after COVID-19 infection, even in older patients, so that IVIG and supportive treatments can be set up promptly, in order to have the best chance of recovery.

Moreover, the comprehensive approach with a geriatrician as the primary attendant may confer to the patients some chances in terms of support therapies to manage the disease while limiting/controlling other geriatric syndromes, such as malnutrition, immobilization, delirium, and pressure ulcers.

We believe that this case study represents a meaningful addition to the existing literature, which may potentially be useful to improve the recognition of GBS related to COVID-19.

### Supplementary Information

**Additional file 1: Appendix 1.** Clinical and diagnostic-therapeutic timeline during the patient’s hospital stay.

## Data Availability

Clinical information and data presented in this manuscript are part of the patient’s medical record, and are available upon request to San Gerardo Hospital ASST Monza Archive.

## Abbreviations

GBS: Guillain-Barré syndrome
AMSAN: Acute motor and sensory axonal neuropathy
COVID-19: Coronavirus disease-2019
ED: Emergency Department
CT: Computed tomography scan
CSF: Cerebrospinal fluid
IVIG: Intravenous immunoglobulin
AGU: Acute Geriatrics Unit
CVC: Central venous catheter
MRI: Magnetic Resonance Imaging

## References

[1] Arcila-Londono X, Lewis RA (2012). Guillain-Barré syndrome. Semin Neurol.

[2] Wachira VK, Peixoto HM, de Oliveira MRF (2019). Systematic review of factors associated with the development of Guillain-Barré syndrome 2007-2017: what has changed?. Tropical Med Int Health.

[3] Sejvar JJ, Baughman AL, Wise M, Morgan OW (2011). Population incidence of Guillain-Barré syndrome: a systematic review and meta-analysis. Neuroepidemiology.

[4] Levin KH (2004). Variants and mimics of Guillain Barré syndrome. Neurologist.

[5] Hughes RA, Swan AV, Raphaël JC, Annane D, van Koningsveld R, van Doorn PA (2007). Immunotherapy for Guillain-Barré syndrome: a systematic review. Brain.

[6] Whittaker A, Anson M, Harky A (2020). Neurological manifestations of COVID-19: a systematic review and current update. Acta Neurol Scand.

[7] Fokke C, van den Berg B, Drenthen J, Walgaard C, van Doorn PA, Jacobs BC (2014). Diagnosis of Guillain-Barré syndrome and validation of Brighton criteria. Brain.

[8] Wittstock M, Benecke R, Zettl UK (2003). Therapy with intravenous immunoglobulins: complications and side-effects. Eur Neurol.

[9] Katz U, Achiron A, Sherer Y, Shoenfeld Y (2007). Safety of intravenous immunoglobulin (IVIG) therapy. Autoimmun Rev.

[10] Zaeem Z, Siddiqi ZA, Zochodne DW (2019). Autonomic involvement in Guillain-Barré syndrome: an update. Clin Auton Res.

[11] Peric S, Berisavac I, Stojiljkovic Tamas O (2016). Guillain-Barré syndrome in the elderly. J Peripher Nerv Syst.

[12] Ellis G, Gardner M, Tsiachristas A, Langhorne P (2017). Comprehensive geriatric assessment for older adults admitted to hospital. Cochrane Database Syst Rev.

[13] Zhou Z, Kang H, Li S, Zhao X (2020). Understanding the neurotropic characteristics of SARS-CoV-2: from neurological manifestations of COVID-19 to potential neurotropic mechanisms. J Neurol.

[14] De Sanctis P, Doneddu PE, Viganò L, Selmi C, Nobile-Orazio E (2020). Guillain-Barré syndrome associated with SARS-CoV-2 infection. A systematic review. Eur J Neurol.

[15] Campana P, Flocco V, Aruta F, Cacciatore F, Abete P (2021). Can aldosterone increase interleukin-6 levels in Covid-19 pneumonia?. J Med Virol.

[16] Sriwastava S, Kataria S, Tandon M (2021). Guillain Barré syndrome and its variants as a manifestation of COVID-19: a systematic review of case reports and case series. J Neurol Sci.

[17] Abu-Rumeileh S, Abdelhak A, Foschi M, Tumani H, Otto M (2021). Guillain-Barré syndrome spectrum associated with COVID-19: an up-to-date systematic review of 73 cases. J Neurol.

[18] Assini A, Benedetti L, Di Maio S, Schirinzi E, Del Sette M (2020). New clinical manifestation of COVID-19 related Guillain-Barrè syndrome highly responsive to intravenous immunoglobulins: two Italian cases. Neurol Sci.

[19] El Otmani H, El Moutawakil B, Rafai MA (2020). Covid-19 and Guillain-Barré syndrome: more than a coincidence!. Rev Neurol (Paris).

[20] Mozhdehipanah H, Paybast S, Gorji R (2020). Guillain–Barré syndrome as a neurological complication of COVID-19 infection: a case series and review of the literature. Int Clin Neurosci J.

[21] Padroni M, Mastrangelo V, Asioli GM (2020). Guillain-Barré syndrome following COVID-19: new infection, old complication?. J Neurol.

[22] Sedaghat Z, Karimi N (2020). Guillain Barre syndrome associated with COVID-19 infection: a case report. J Clin Neurosci.

[23] Toscano G, Palmerini F, Ravaglia S (2020). Guillain-Barré syndrome associated with SARS-CoV-2. N Engl J Med.

[24] Manganotti P, Bellavita G, D'Acunto L (2021). Clinical neurophysiology and cerebrospinal liquor analysis to detect Guillain-Barré syndrome and polyneuritis cranialis in COVID-19 patients: a case series. J Med Virol.

[25] Finsterer J, Scorza FA (2021). Guillain-Barre syndrome in 220 patients with COVID-19. Egypt J Neurol Psychiatr Neurosurg.

