# Supplementary material for: Guillain-Barré syndrome AMSAN variant in a 90-year-old woman after COVID-19: a case report
Source: BMC Geriatr. 2023 Mar 1;23:114. doi: 10.1186/s12877-023-03833-1 (PMC9975860; doi:10.1186/s12877-023-03833-1)
Supplement: Supplementary file 1 — Additional file 1: Appendix 1. Clinical and diagnostic-therapeutic timeline during the patient’s hospital stay. [file 12877_2023_3833_MOESM1_ESM.docx]

**SUPPLEMENTARY MATERIAL**

**Appendix 1.** Clinical and diagnostic-therapeutic timeline during the patient’s hospital stay

|  | First days of December 2020 | 12/26/2020 | 1/12/2021 | 1/13/2021 | 1/15/2022 | 1/15/2022 | 1/16/2022 | 1/27/2021 |
| --- | --- | --- | --- | --- | --- | --- | --- | --- |
| **Location** | Home | ED | ED | AGU | AGU | AGU | AGU | Discharge ah home |
| **Sign and Symptoms** |  | Loss of appetite | Fatigue, worsening gait and leg strength, dysphonia, dysarthria, dysphagia | Dysphonia, dysarthria, dysphagia, symmetric weakness in the upper limbs and asymmetric in lower limbs, pressure sore | Dysphonia, dysarthria, dysphagia, symmetric weakness in the upper limbs and asymmetric in lower limbs | Uncontrolled hypertension, acute urine retention; persisted neurological symptoms | Shortness of breath, hypoxemia | Slight improvement of dysphonia and dysarthria; dysphagia and motor deficit unchanged, urinary incontinence, pressure sore |
| **Disease** | SARS-CoV-2 infection | Dehydration | GBS versus encephalopathy | GBS versus encephalopathy | GBS AMSAN type | Autonomic dysfunctions | Aspiration pneumonia |  |
| **Diagnostic evaluation** | Nasopharyngeal swab test | Laboratory test | Laboratory test, CFS, cerebral CT scan | Laboratory test | EMG | - | Chest X-rays, laboratory test |  |
| **Therapy** | Paracetamol | Parenteral hydration | Acyclovir, ampicillin, parenteral hydration | Acyclovir, ampicillin parenteral hydration | IVIG, parenteral nutrition | IVIG, urinary catheter, antihypertensive therapy | IVIG, parenteral nutrition Metronidazole | Home care support (parenteral nutrition, pressure wound dressing, urinary catheter, CVC), physiotherapy |

Abbreviations: CSF = cerebrospinal fluid; EMG = electromyography; ED = Emergency Department: GBS = Guillain-Barré syndrome; AMSAN = acute motor and sensory axonal neuropathy; IVIG = intravenous immunoglobulin; AGU = Acute Geriatrics Unit; CT = computed tomography; CVC = central venous catheter
